# A Review on Health-Promoting, Biological, and Functional Aspects of Bioactive Peptides in Food Applications

**DOI:** 10.3390/biom11050631

**Published:** 2021-04-23

**Authors:** Seyed Hadi Peighambardoust, Zohreh Karami, Mirian Pateiro, José M. Lorenzo

**Affiliations:** 1 Department of Food Science, College of Agriculture, University of Tabriz, Tabriz 5166616471, Iran; peighambardoust@tabrizu.ac.ir (S.H.P.); zohrehkarami92@gmail.com (Z.K.); 2 Centro Tecnológico de la Carne de Galicia, Rúa Galicia No. 4, Parque Tecnológico de Galicia, San Cibrao das Viñas, 32900 Ourense, Spain; mirianpateiro@ceteca.net; 3 Área de Tecnología de los Alimentos, Facultad de Ciencias de Ourense, Universidad de Vigo, 32004 Ourense, Spain

**Keywords:** peptides, technological properties, antioxidant activity, ACE-inhibitory activity, hypocholesterolemic activity, functional food, human health

## Abstract

Food-derived bioactive peptides are being used as important functional ingredients for health-promoting foods and nutraceuticals in recent times in order to prevent and manage several diseases thanks to their biological activities. Bioactive peptides are specific protein fractions, which show broad applications in cosmetics, food additives, nutraceuticals, and pharmaceuticals as antimicrobial, antioxidant, antithrombotic, and angiotensin-I-converting enzyme (ACE)-inhibitory ingredients. These peptides can preserve consumer health by retarding chronic diseases owing to modulation or improvement of the physiological functions of human body. They can also affect functional characteristics of different foods such as dairy products, fermented beverages, and plant and marine proteins. This manuscript reviews different aspects of bioactive peptides concerning their biological (antihypertensive, antioxidative, antiobesity, and hypocholesterolemic) and functional (water holding capacity, solubility, emulsifying, and foaming) properties. Moreover, the properties of several bioactive peptides extracted from different foods as potential ingredients to formulate health promoting foods are described. Thus, multifunctional properties of bioactive peptides provide the possibility to formulate or develop novel healthy food products.

## 1. Introduction

**Figure 1:**
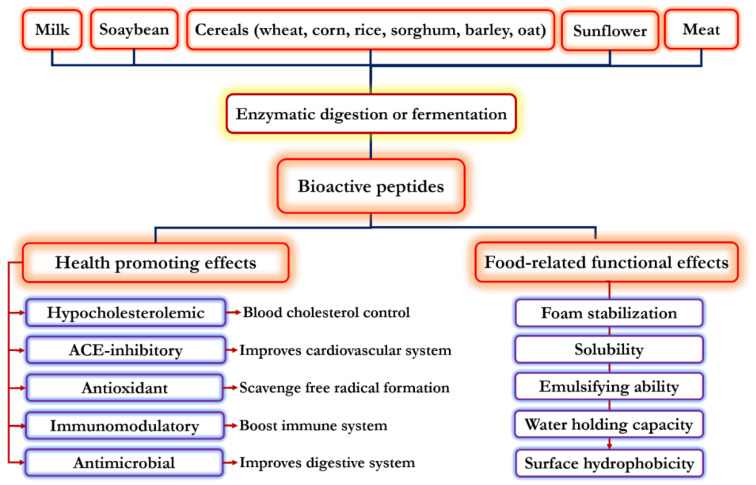
Health-promoting and functional properties of bioactive peptides. ACE, angiotensin-I-converting enzyme.

Recently, there has been more attention paid to natural source health-promoting ingredients such as phenolic compounds and bioactive peptides. Particularly, a large range of bioactive peptides were identified from various food products. Bioactive peptides can be derived from animal and plant origins including soybean, cereals germ, potato, nuts, dairy products, egg, and meat proteins [1,2,3,4]. In the native sequences, these peptides do not show biological activity; however, they can be activated and exhibit biological activity by enzymatic, chemical, and microbial hydrolysis [5]. Enzymatic hydrolysis can be regarded as the most effective method to produce active peptides [1,2], which can be easily absorbed in intestinal tract and directly entered to the blood stream. This ensures their in vivo bioavailability and physiological functionality in the body [6]. Bioactive peptides are considered as important health-promoting food ingredients with the possibility of improving human health and preventing disease. In many studies, the antihypertension, antithrombotic, anti-cancer, antimicrobial, antioxidant, immunomodulatory, and opioid agonists or antagonists properties of these peptides have been reported [7]. Different factors such as processing conditions, protein source, amino acids’ sequence and composition, molecular weight (Mw) and charge distribution, pH, and certain chemical treatments can directly affect the functionality of bioactive peptides [8]. Thus, this review highlights novel perspectives of potential bioactivity of peptides originating from most food products, with special attention on various factors governing their functionality and biological aspects in medical biochemistry. A summary of health-promoting effects and food-related functional properties of bioactive peptides is illustrated in Figure 1.

## 2. Functional Properties of Food-Derived Bioactive Peptides

### 2.1. Solubility

The solubility of molecules depends on their molecular size. When proteins are hydrolysed to smaller peptides, their solubility will increase. The polarity of amino acids in the peptide structure can influence hydrophile to hydrophobic balance, which, in turn, can influence their solubility. Thus, the molecular size and solubility of peptides are governed by the enzymatic hydrolysis conditions and the existence of polar and ionisable groups in their structure [9]. For instance, peptides with low molecular weight (Mw) obtained from myofibrillar proteins have polar residues, which can lead to the formation of hydrogen bonds, increasing their solubility. Smaller peptides obtained from a higher degree of hydrolysis are more soluble. pH is also another factor that affects biopeptides’ solubility. Generally, bioactive peptides are more soluble at their isoelectric point (pI), as pH affects the net charge in side chain groups of amino acids in peptides and their surface hydrophobicity. It is reported that peptides’ solubility is increased when pH varies from their pI point and hydrophobic interactions, and aggregation behaviour in peptides is influenced by their surface hydrophobicity [10].

### 2.2. Emulsifying Properties

Bioactive peptides and protein hydrolysates can show surface-active properties owing to their hydrophilic and hydrophobic groups, thus promoting the stabilization of oil-in-water emulsions [11]. The mechanism behind emulsifying properties of the peptides relies on surface interaction of peptides on oil droplets during the homogenization process, leading to creation of a repulsive surface layer that can avoid oil droplets’ coalescence [9,12]. It has been shown that emulsifying activity of peptides and the resulting emulsion stability has been negatively affected by degree of hydrolysis (DH) [13]. Lower DHs lead to strong emulsifying activity in peptides and hydrolysates [13]. On the other hand, higher DHs resulting from excessive hydrolysis conditions can lead to the loss of emulsifying properties, owing to the fact that low Mw peptides are not amphiphilic enough to show emulsifying activities [13]. At low DH conditions, larger peptides are obtained, which can effectively decrease the interface tension because of the fact that they can be easily unfolded and reoriented at the interface of oil and water droplets. In contrast, at higher DHs, peptides and hydrolysates can be rapidly diffused and adsorbed at the interface [13].

### 2.3. Foaming Ability

Similar to emulsifying properties, the foaming ability of hydrolysed peptides is affected by their molecular size and degree of hydrolysis. Peptides with high Mw tend to positively affect foam stability. Generally, denatured and hydrolysed proteins have a good foaming ability thanks to the structural changes and unfolding of these hydrolysates, which can easily migrate to the air–water interface and be rearranged at the interface [13]. It is reported that the foam formation ability of proteins can be enhanced by exposing hydrophobic residues in their structure, and by decreasing their surface tension. Hydrolysates with an unfolded structure and more hydrophobic regions can be easily adsorbed at the air–water interface, leading to stronger foaming properties [14]. Reversely, at excessive hydrolysis conditions (higher DHs), foaming stability is reduced because peptides with low Mw do not have enough strength to maintain strong foams.

As mentioned previously, surface hydrophobicity of peptides is also influenced by pH. Net charge of peptide residues may affect the protein adsorption at the air–water interface, which will influence their foaming ability. For instance, acidic conditions negatively affect the foaming properties of hydrolysates [13]. As a result of net charge and pH variations, protein solubility can be varied, which may play an important role in the foaming behaviour of protein hydrolysates; higher foaming ability can be reached at the lowest solubility [13].

### 2.4. Water Holding Capacity

Water holding capacity (WHC) is a key factor in determining the functionality and yield of protein products [12]. WHC can be related to mechanical properties (elasticity, plasticity) and flow behaviour of food materials. Protein hydrolysates from seal meat [15] and bovine skin gelatin hydrolysate [5] were found to improve WHC of meat products. In addition, WHC can be influenced by concentration of the peptides, pH, and the type of enzymes used for hydrolysis. It has been reported that low Mw peptides show more WHC than high Mw ones owing to their smaller peptides with more hydrophilic nature. Protein hydrolysates obtained from fish showed better solubility within a wide ionic and pH range. These hydrolysates tolerated high temperatures with no precipitating [16]. It was also shown that incorporating fish protein hydrolysates to food products contributed to their WHC and improved textural attributes, gel forming ability, whipping, and emulsifying properties.

### 2.5. Hydrophobicity

Hydrophobicity of bioactive peptides plays a key role in their physical interactions with biological molecules, which is important to determine their mechanism of action and physiological functions [17]. In this regard, the surface hydrophobicity is involved in the formation and/or maintenance protein three-dimensional structures and interactions. Some examples are the following: the ability of peptides for binding to the membranes of cells, protein–protein interaction, and complex formation with biological molecules. Upon protein hydrolysis, the folded and globular structures of proteins become unfolded. This may lead to exposure of the hydrophobic regions of the protein, which were previously hidden in the native structure of protein [18]. It has been reported that the hydrolysis of kidney bean protein leads to an increase in the surface hydrophobicity of the resulting hydrolysates compared with that of the original protein. The increased hydrophobicity may be related to exposed hydrophobic residues of proteins [18].

## 3. Physiological Properties of Food-Derived Bioactive Peptides

**Figure 2:**
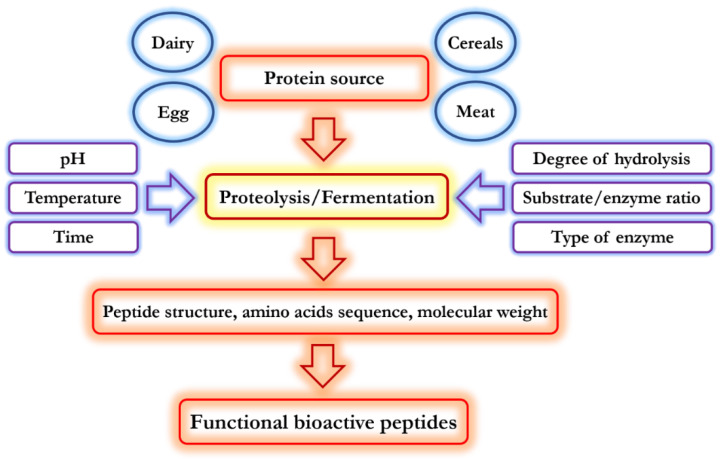
Effect of different operational conditions and parameters on biological and functional properties of bioactive peptides.

Bioactive peptides show health-promoting functions of different target organs such as heart, bone, and gastrointestinal tract [19]. They can also improve immune defence functionality, control stress, and improve human mood [20]. Various factors such as protein type and source, protein pre-treatment, enzyme type, and the conditions of proteolysis can alter the functionality of hydrolysates and bioactive peptides [21,22]. In addition, functional and physiological properties of bioactive peptides such as antioxidant, antimicrobial, and angiotensin-I-converting enzyme (ACE) inhibition activities can be effectively influenced by the structural properties and the types and sequence of their amino acids. Meanwhile, these peptides can impart in foods functional properties by contributing to WHC, textural attributes, gel forming ability, whipping, and emulsifying properties [23]. Consequently, different factors influence peptides biological functions including the source of protein, operational conditions (time, temperature, hydrostatic pressure, ultrasound, pulsed electric fields, and so on) used to produce peptides, as well as proteolysis conditions (degree of hydrolysis, substrate-to-enzyme ration, enzyme type, pH, temperature). These parameters are shown in Figure 2.

### 3.1. Bioactive Peptides with Antioxidant Activity

Protein hydrolysates obtained from different sources show stronger antioxidant properties than their corresponding purified peptides [24]. There are many reports that describe antioxidant peptides from different food sources such as peanut protein [25], wheat germ [2,3], rice bran [26], sunflower [27], alfalfa leaf [28], corn gluten [29], frog skin [30], tuna liver [31], porcine liver [32], egg-yolk [33], milk and soymilk kefir [34], algae protein waste [35], and buckwheat protein [36]. The biological activity of these peptides, including antioxidant properties, is influenced by the processing conditions used in protein isolation, the protein type, the extent of hydrolysis, protease enzyme type, the structure of peptide, Mw of peptides, and peptide concentration, as well as proteolysis conditions such as substrate-to-enzyme ratio, pH, processing time, and temperature [29,37], as shown in Figure 2. A peptide’s particular structure can influence its antioxidant behaviour. In this regard, it was reported that some amino acids exhibit higher antioxidant characteristics in the form of dipeptides. However, other findings suggested that the conformation structure of proteins influences the antioxidant potential of the resulting peptides by proteolysis. Moreover, hydrolysis conditions are important in antioxidant activity of protein hydrolysates [38]. Table 1 summarizes some of the existing studies on the effect of different enzymes, applied on some protein sources, in the production of antioxidant peptides with identified amino acid sequences.

#### 3.1.1. Milk-Derived Bioactive Peptides

Milk proteins are a source of biologically active peptides [56]. Milk peptides from casein and whey proteins have been shown to be a strong source of antioxidant peptides. Milk-derived peptides with antioxidant activity contain 5–11 amino acids, with hydrophobic residues like tryptophane, tyrosine, histidine, and proline being among them. Aromatic amino acid-rich whey protein has a higher antioxidant capacity than simple amino acid [57]. The antioxidant activity of peptides can be influenced by amnio acid content and position within the chain. The amino acids histidine, tyrosine, lysine, phenylalanine, valine, and cysteine were significantly higher in whey protein-derived peptides than in WPI hydrolysate, suggesting that these amino acids are essential for antioxidant activity [58]. The hexapeptide YFYPEL is generated by pepsin hydrolysis of bovine casein and scavenges superoxide radicals [51]. Enzymatic peptides found in the hydrolysate of whey proteins by Corolase or other commercial proteases were also found to have a high capacity for scavenging free radicals. A total of 42 peptide fragments were identified containing the sequence WYSLAMAASDI, showing the highest activity. The content of specific amino acids, in particular high quantities of histidine (which has peroxyradical trapping and chelating powers) and hydrophobic amino acids (which increase the accessibility of peptides to hydrophobic targets), has been attributed to the potency of antioxidant activity of bioactive peptides [59]. Shazly et al. [60] used Alcalase and trypsin to hydrolyze buffalo and bovine caseins, resulting in novel antioxidant peptides. Ultrafiltration (UF) and Reversed-Phase High Performance Liquid Chromatography (RP-HPLC) were used to purify the casein hydrolysates. The fractions with molecular weight smaller than 1 kDa as well as hydrolysate produced by Alcalase for buffalo casein showed higher antioxidant activity than that obtained by trypsin. They proved that trypsin hydrolysate contained a high amount of hydrophobic amino acids, while Alcalase hydrolysate consisted mainly of serine, arginine, alanine and leucine. The antioxidant peptides identified by LC MS/MS were RELEE, MEDNKQ and TVA, EQL in buffalo casein hydrolysates produced by trypsin and Alcalase, respectively. *K-Casecidin* is a pentapeptide with antioxidant activity corresponded to bovine *k*-casein *f*(17–21) [61]. It was also shown that caseins originated from yak milk could produce peptides with antioxidant properties, enabling them to be considered as bioactive substances in value-added functional foods [62,63,64]. Soy milk and its fermented products are attractive sources of bioactive peptides with great biological activity [65]. In this regard, soy milk kefir has shown considerable antimutagenic and antioxidant properties [34]. It was also shown that hydrolysis of soy protein with microbial proteases can produce hydrolysates or biopeptides with strong antioxidant activities [66]. Accordingly, these peptides may be considered as alternative natural antioxidants in food products and can delay rancidity owing to the lipid oxidation [65,67].

#### 3.1.2. Meat-Derived Bioactive Peptides

Meat by-products can also be used to produce functional ingredients rich in proteins [68]. Most of the peptides are extracted from bovine (blood, kidney, liver, lung, pancreas) and porcine (appendix, blood, colon, heart, liver, lung, pancreas, and rectum) sources [69,70,71]. However, other sources such as poultry by-products (bones, feet, and skin) have emerged owing to regulation, religions restrictions, or culture traditions [72]. Several types of enzymes have been tested for their extraction such as Alcalase, bromelain, flavourzyme, papain, or pepsin, with papain, pepsin, and Alcalase being those that reported the most successful results for releasing antioxidant biopeptides from animal tissues [73,74]. In this regard, López-Pedrouso et al. [43] found that the use of Alcalase in the enzymatic hydrolysis of porcine liver displayed better results than those obtained with bromelain, flavourzyme, and papain. The peptides released were smaller and showed a peptidomic pattern more differentiated than those found with the other enzymes. Furthermore, using Sequential Window Acquisition of all Theoretical Mass Spectra (SWATH-MS), a data-independent acquisition method that complements conventional mass spectrometry-based proteomic techniques, the researchers were able to classify and quantify peptides derived from trypsinogen, ferritin, and uncharacterized protein (Table 1). In the same way, the application of Alcalase for the enzymatic hydrolysis of porcine liver resulted in hydrolysates with antioxidant activity [32]. The identified peptides (Table 1) were significantly correlated with antioxidant assays (2,2-Diphenyl-1-picrylhydrazyl radical scavenging activity (DPPH), 2-2’-Azino-di-[3-ethylbenzthiazoline sulfonate] radical scavenging activity (ABTS), ferric reducing antioxidant power (FRAP), oxygen radical absorption capacity (ORAC)) at different times of hydrolysis. Their activity is probably linked to the presence of hydrophobic (leucine, valine, and isoleucine) and aromatic (tyrosine and phenylalanine) amino acids. This is in accordance with the fact that amino acid content determines their biological activity [75].

#### 3.1.3. Marine-Derived Bioactive Peptides

Seafood is also an important source of derived antioxidant peptides [76]. In this regard, many researches have produced antioxidant peptides from protein hydrolysates of different marine sources such as blue mussel [77], jumbo squid [78], tuna [79], oyster [30], scad [80], cod [16], yellow stripe trevally [9], and microalgae [35]. They showed that these bioactive peptides can scavenge free radicals and remove reactive oxygen, thus leading to preventing lipid peroxidation by interrupting the radical chain reactions [35]. Mendis et al. [78] isolated a marine bioactive peptide from jumbo squid and investigated the inhibition of lipid peroxidation by the obtained peptide. It was shown that peptide antioxidant activity was much higher than that of α-tocopherol and was similar to BHT. In addition, gelatin peptides are composed mainly of hydrophobic amino acids, which causes their higher emulsifying ability. Thus, it is possible that bioactive peptides derived from marine gelatin exhibit higher antioxidant activity among other antioxidant peptide sequences [78]. In fact, gelatin-derived peptides have a unique amino acid arrangement with a repeating sequence of Gly-Pro-Ala that was specifically associated with antioxidant properties [81]. Moreover, anticoagulant marine bioactive peptides were separated from marine organisms such as marine echiuroid worm [82] and blue mussel [83].

Joshi et al. [84] used thermolysin and pepsin to investigate the effect of peptide availability on antioxidant potency from by-catch shrimp (*Oratosquilla woodmasoni*) waste. The peptide with molecular weight 431 Da with the sequence of NGVAA was found from the active purified fraction as antioxidant peptide. In terms of the enzyme type effect, when fish protein from *Selaroides leptolepis* was hydrolyzed with Alcalase, antioxidant peptides were generated at a low DH of 5%, whereas Flavourzyme 500L hydrolysates performed better at a DH of 25% [13]. The antioxidant activity of seaweed as investigated by Heo et al. [85] showed that the antioxidant ability of the Alcalase hydrolysates of *Sargassum horneri* was dose-dependent and thermally stable. Furthermore, Harnedy et al. [86] used the food-grade enzyme Corolase PP to generate an enzymatic hydrolysate of *Palmaria palmata*. The peptide SDITRPGNM was reported as having the highest oxygen radical absorption capacity (ORAC) and ferric reducing antioxidant power (FRAP) activity after purification using RP-HPLC.

#### 3.1.4. Plant-Derived Bioactive Peptides

Because of the high production quantity and low unit costs, plant by-products are a reasonable alternative to animal sources in bioactive peptide production. Proteins present in cereals have been recognized as positive contributors to biological functions such as antioxidant activities [87]. Cermeño et al. [40] isolated three peptides, IPY, LPY, and YPLP, from brewers’ spent grain by-products with antioxidant activity, in which this activity can be attributed to the tyrosine in their sequences. In the study of Xie et al. [88], mung bean protein was hydrolyzed by Alcalase and the fractions with less than 3 kDa showed the highest antioxidant activity (DPPH, hydroxyl, and superoxide radical scavenging activity and metal chelating assay). The results showed that these fractions had more hydrophobic and aromatic amino acids and their secondary structure was composed of α-helix, β-sheet, and irregularly coiled. Three peptides with the sequences of FGER, FDRR, and FGERR were identified as antioxidant peptides from potato protein hydrolysates [45]. The peptides had phenylalanine at the N-terminus and arginine at the C-terminus, which could be due to the specificity of the enzyme. These peptides showed antioxidative activity according to the methods of β-carotene decolorization and ferric thiocyanate. Karami et al. [3] hydrolysed wheat germ by different peptidases and introduced the KELPPSDADW peptide from pepsin, the peptides SGGSYADELVSTAK and MDATALHYENQK from proteinase K, and the peptide GNPIPREPGQVPAY from Alcalase hydrolysates as radical scavengers. They proved that the presence of aromatic residues (such as tyrosine (Y), tryptophan (W), histidine (H), and methionine (M)) was effective on antioxidant activity owing to their particular structural features. In studies, peptides obtained from plant by-products such as sesame bran (sulphur-containing methionine and cysteine) [89], wheat bran (NL, QL, FL, HAL, AA-VL, and AKTVF) [90], sunflower seeds (copper-chelating peptides) [27], rapeseed (WDHHAPQLR) [48], and hemp seeds (WVYY and PSLPA) [91] were reported to contain mentioned amino acids in the structure of bioactive peptides and showed remarkable antioxidant properties. Bioactive peptides derived from protein-rich plant by-products may be used in the production of functional foods. The amino acid type, sequence, and molecular weight all influence the functional properties and biological activities of bioactive peptides.

### 3.2. Bioactive Peptides with Angiotensin I-Converting Enzyme (ACE) Inhibitory Activity

Angiotensin I-converting enzyme (ACE) is responsible for the conversion of angiotensin I to the potent vasoconstrictor angiotensin II, as well as the inactivation of the vasodilator bradykinin, which results in blood pressure regulation. Compounds with ACE inhibition effect can be used to control blood pressure in patients with hypertensive symptoms [92]. Certain synthetic ACE inhibitors such as ramipril, captopril, lisinopril, alacepril, and enalapril have been extensively applied for the effective treatment of human hypertensive symptoms and heart diseases [93]. However, these synthetic chemical inhibitors may show different side effects such as diarrhea, allergy, cough, taste disorders, and skin rashes [94]. Thus, recently, there has been a trend to use natural ACE inhibitors instead of those synthetic drugs. Nevertheless, in some cases, natural ACE inhibitors such as bioactive peptides may show lower efficiency than their synthetic competitors. In this regard, bioactive peptides hydrolysed from protein sources could be applied in the initial treatment of the hypertension symptoms in the individuals [29]. Peptides with both antioxidant and ACE inhibition properties have normally hydrophobic amino acids, which are able to interact with target enzymes or free radicals [95]. The structure of peptides is essential in ACE inhibition. The main characteristics of ACE inhibitory peptides are chain length, peptide structure, and sequences. ACE-inhibitory peptides are usually short chain peptides with 2–12 amino acids, and crystallography studies have shown that large peptides cannot bind to ACE active sites. Long chain peptides, on the other hand, may have ACE-inhibitory activity in certain cases, as the amino acid form may be more significant than the peptide duration. This may be linked to amino acid composition, as peptides with highly acidic amino acids (Asp and Glu) have a net negative charge that chelates zinc atoms, which are needed for enzyme activity [96]. The C- and/or N-ends of ACE-inhibitory peptides are usually made up of distinct amino acid residues. Tyrosine, phenylalanine, tryptophan, proline, lysine, isoleucine, valine, leucine, and arginine have been shown to have a significant effect on ACE binding in peptides. The presence of positively charged amino acids at the C-terminus has also been found to affect the inhibitory effects of peptides. The behavior of ACE is influenced by both the charged amino acids and the number of amino acids in the bioactive peptide. ACE inhibitors come in a variety of sequences, ranging from dipeptides to oligopeptides. Amino acids with bulky and hydrophobic side chains make up these dipeptides. An aromatic amino acid was found at the first residue, a positively charged amino acid at the second residue, and a hydrophobic amino acid at the third residue in the case of tripeptide. Tyrosine and cysteine are in the first place; histidine, tryptophane, and methionine are in the second position; Ile, Leu, Val, and Met are in the third position; and tryptophane is in the fourth position of ACE-inhibitory tetrapeptides [97]. Table 2 indicates some antihypertensive peptides derived from food sources.

#### 3.2.1. Milk-Derived Bioactive Peptides

As previously commented, bioactive peptides hydrolysed from milk proteins have important roles in human health [104]. Milk caseins are a good source of bio-functional peptides. In this regard, peptides with ACE-inhibitory activity were obtained from the sequence 60-70 of β-casein [105]. In another study, ACE-inhibitory peptides such as “valine–arginine–tyrosine–leucine” hydrolysed from cow milk proteins [106] and goat milk hydrolysate [107] have been isolated, which exhibited antihypertensive effects [108]. Erdmann et al. tested the antihypertensive and ACE-inhibitory activity of a peptide isolated from the casein hydrolysate obtained from goat milk, showing that peptides with IC_50_ values of 316–354 µmol/L exhibited antihypertensive activity in rats [6]. Fermented bovine milk can also be source of peptides with ACE-inhibitory activity including Val-Pro-Pro corresponding to *β*-casein *f*(84–86) and Ile-Pro-Pro to *β*-casein *f*(74–76) [109]. The same happens with human milk, as a potent ACE-inhibitory peptide was also determined in it, corresponding to the β-casein fragment *f*(125–129) with the sequence HLPLP [63]. Therefore, owing to use of commercial dairy and probiotic starter cultures with potent proteolytic activity in the production of fermented dairy products, it is possible to obtain bioactive peptides resulting from proteolytic reactions [110].

Generally, most of the peptides are released during cheese ripening, exhibiting biological activities. In this regard, calcium phosphopeptides have been detected in Comte’ and Cheddar cheese [111]. The presence of these peptides is dependent on the ripening stage of the cheese. Excessive proteolysis as a result of very extended ripening stage may reversely decrease the amount of bioactive peptides or decrease their bio-functionality [112]. This suggests that bioactive peptides in cheese increase with cheese ripening stage, but decline upon excessive proteolysis, and it could be assumed that ACE-inhibitory ability of peptides in dairy products with a low degree of proteolysis, such as yogurt and fresh cheese (i.e., quark), is low. Changing a trans to a cis-form of Pro in an ACE-inhibitory peptide’s C-terminal position can cause major changes in the enzyme’s interaction. DKIHP (-casein f47–51), an ACE-inhibitory peptide obtained from Manchego cheese, was analyzed using two separate preparations by Gómez-Ruiz et al. [113]. One preparation with a special conformer containing trans-Pro showed strong ACE inhibitory activity. The second one had three different conformers, two with trans-Pro and one with cis-Pro, and had a lower ACE-inhibitory activity than the first. Proteolytic strains of the LAB species *L. helveticus*, *L. casei*, *L. plantarum*, *L. rhamnosus*, *L. acidophilus*, and *Lactococcus lactis*, as well as the two species used in conventional yoghurt processing, were used to produce fermented milks containing an especially large number of peptides.

#### 3.2.2. Meat-Derived Bioactive Peptides

Meat products are high in functional biopeptides, which are health-promoting compounds. ACE-inhibitory activity in some naturally formed peptides was calculated empirically using consecutive fractionation steps to isolate the most active fractions, and thus classify the peptides by MS in tandem in several studies of meat products. As peptide structure plays an important role in ACE inhibition, some of the known peptides were chosen as potential bioactive based on their duration, amino acid composition, and amino acid location in the sequence [114]. ACE inhibitory properties of peptide extracts from Spanish Teruel, Italian Parma, and Belgian dry-cured ham have recently been investigated [114]. The antihypertensive activity of the most active fractions and peptides found in Spanish dry-cured ham was also tested in vivo using the SHR model (Spontaneously Hypertensive Rats). After 8 h of ingestion, fractions from size-exclusion chromatography with molecular masses less than 1700 Da demonstrated the strongest antihypertensive activity, with a reduction in systolic blood pressure (SBP) of 38.38 mmHg in spontaneously hypertensive rats [115], whereas after 8 h of administration in SHR, peptide AAATP with an in vitro IC_50_ value of 100 M showed a decrease in SBP of 25.62 mmHg [116]. Furthermore, using a combination of an in silico model and a conventional in vitro method, fractions obtained after GI digestion of Parma dry-cured ham of 18 and 24 months of curing were analyzed to identify ACE-inhibitory peptides, resulting in the identification of several small peptides such as LGL and SFVTT [117]. The muscle of the black-bone silky fowl (*Gallus gallus domesticus Brisson*) was hydrolyzed with Alcalase and papain, and peptides LER and GAG with ACE inhibitory activity were discovered [118]. Choe et al. [119] used thermolysin to identify ACE inhibitory peptides in beef *M. longissimus*. In addition, the thermolysin hydrolysate was fractionated into seven different groups. As fraction V had the highest ACE inhibitory activity, it was further sub fractionated to obtain unique peptides. The peptides LSW, FGY, and YRQ had the highest ACE inhibitory activity and had higher concentrations than the other peptides studied. These findings indicated that, when thermolysin is used to lyse proteins, bioactive peptides with strong ACE inhibitory activity are generated.

#### 3.2.3. Marine-Derived Bioactive Peptides

The hydrolysis conditions, substrate protein, and form of peptidase all affect the development of ACE inhibitory peptides. Enzymic digestion of a marine protein substrate yielded ACE-inhibitory protein hydrolysates, according to research. The peptide GDLGKTTTVSNWSPPKYKDTP has been isolated from tuna frame protein hydrolysate by Lee et al. [120], which exhibited a strong suppressive effect on systolic blood pressure of spontaneously hypertensive rats, and this antihypertensive activity was similar to that shown by captopril. Other possible sources of bioactive peptides are by-products of cannery sardine. Two novel ACE inhibitory tri-peptides, FTY and FSY, were identified in the hydrolysate obtained from Northern shrimp (*Pandalus borealis*) [121]. As both terminal residues are identical and both intermediate residues (serin and threonine) have R-groups containing hydroxyl, the two peptides are very similar. The only difference is that FTY is significantly more bulky than FSY because the R-group of Thr contains a free methyl in addition to the hydroxyl. As FSY is around 30 times more active than FTY, the results show that this small difference has a significant effect on ACE inhibitory activity. Sardine muscle hydrolysates with alkaline protease have been found to have ACE-inhibitory activity, with the peptides KW, RVY, and MY being known as antihypertensive peptides [122].

#### 3.2.4. Plant-Derived Bioactive Peptides

Vieira and Ferreira [123] obtained hydrolysates with ACE-inhibitory activity (IC_50_ of 164 μg/mL) by enzymatic hydrolysis using proteases obtained from brewer’s spent yeast. The authors found that low-Mw peptides present higher ACE-inhibitory activity. The same trend was observed by Tian et al. [124] in peptides obtained from yak (*Bos grunniens*) skin. The higher ACE-inhibitory activity was obtained in the fraction with low Mw (<3 kDa) obtained from the hydrolysis with proteinase K and Alcalase (IC_50_ of 0.65 mg/mL). Moreover, the amino acid composition of the obtained peptides could also be a key factor. In this regard, sardine protein hydrolysates showed an important content of hydrophobic amino acids (proline, leucine, glycine, isoleucine, phenylalanine, and valine). In fact, ACE-peptides usually have a proline residue at the carboxyl terminal end [125].

Cermeño et al. [40] showed the brewer’s spent grain by-products hydrolysates obtained from Alcalase and flavourzyme have remarkable biological activities. The results of purification and fractionation by UPLC-ESI-MS/MS identified peptides with sequences of IPY, IPLQP, LPLQP, APLP, VPIP, IPVP, PLVP, IPVP, YPLP, LPY, and YPLP. Among these peptides, APLP, IPLQP, IPVP, and VPIP exhibited good ACE inhibitory activities. They reported that the presence of P in the sequence is related to good ACE-inhibitory activity. Thus, the peptides LPLQP and IPLQP showed the highest ACE-inhibitory activity and the peptides APLP, VPIP, IPVP, and PLVP showed a moderate activity. Therefore, the structure of amino acids could be a good prognosticator of peptides’ bioactivity. They also investigated the effect of leucine and isoleucine on peptide bioactivity and concluded that isoleucine at the N-terminal position (for example, peptide IPVP) show good ACE-inhibitory activity, while leucine at the same position (for example, peptide LPVP) has no ACE-inhibitory activity. This difference can be attributed to the structural properties of leucine and isoleucine. Moreover, Connolly et al. [126] identified two peptides ILDL and ILLPGAQDGL with high ACE-inhibitory activity (96.4 and 306 μM, respectively). Another two ACE-inhibitory peptides with the sequences IVY and LLDL were isolated using Alcalase from brewer’s spent grain hydrolysates, which displayed IC_50_ of 80.4 and 96.4 μM, respectively [100].

Li et al. [98] studied the hydrolysis of mung bean protein using Alcalase and MALDI-TOF analysis and showed that KDYRL, VTPALR, and KLPAGTLF peptides displayed antihypertensive activity with values of IC_50_ of 26.5, 82.4, and 13.4 µM, respectively. Sonklin et al. [99] hydrolysed mung bean protein using bromelain, and the hydrolysates were divided into four fractions based on their Mw (i.e., Mw < 1 kDa, Mw = 1–5 kDa, Mw = 5–10 kDa, and Mw > 10 kDa). The results showed that the fraction with Mw < 1 kDa showed the highest ACE-inhibitory activity. In this regard, it was reported that peptides with low Mw can attach to active sites of ACE more easily than peptides with high Mw, and showed the highest ACE inhibitory activity [127]. Peptides such as LPRL, YADLVE, LRLESF, HLNVVHEN, and PGSGCAGTDL were identified from the fractions of Mw < 1 kDa in mung bean and showed antihypertensive activity. Proteinase K is also useful to obtain bioactive peptides with ACE-inhibitory properties. In this regard, Karami et al. [2] isolated the antihypertensive peptides IGGIGTVPVGR, SGGSYADELVSTAK, MDATALHYENQK, VDSLLTAAK, and VALTGDNGHSDHVVHF from wheat germ using proteinase K. They attributed this feature to the presence of basic residues such as R and K, or to aliphatic residues such as leucine (L), valine (V), alanine (A), or proline (P), or aromatics such as W, Y, or phenylalanine (F).

The biological activity of peptides is affected by their structure, amino acid composition, and the type of amino acid positions such as N-terminal and C-terminal. LRLESF peptide showed the strong antihypertensive activity, whereas LPRL peptide had weak antihypertensive activity [128]. The presence of leucine in all peptides shows positive effects on ACE inhibitory activity; especially, the position of leucine at N-terminal and phenylalanine at the C-terminal is reported to have strong ACE inhibition activity [129]. However, when leucine is located at the C-terminal (i.e., in LPRL peptide), it shows no strong activity [100]. The peptide YADLVE exhibits the strongest renin inhibitor because it has tyrosine at N-terminal and glutamic acid at the C-terminal, and this characteristic is responsible for the renin-inhibitory activity of peptides.

### 3.3. Bioactive Peptides with Hypocholesterolemic Activity

Hypocholesterolemic effect of bioactive peptides has been shown in several protein sources including soy, whey, and fish [130]. Soy protein hydrolysates and peptides can reduce blood total cholesterol levels more effectively than their parental protein. Bioactive peptides obtained from hydrolysis of soy glycinin, LPYPR, and IAVPGQVA were reported to reduce serum cholesterol [131]. The exact mechanism of hypocholesterolemic effect for bioactive peptides is not clear, but evidence shows that biopeptides can improve blood lipid profiles by changing atherogenic plasma to cardioprotective plasma profile [6]. It is also reported that several possible mechanisms, such as induction of LDL receptor expression, enhancement of bile acid synthesis, and decline in steroid absorption from the intestine, are responsible for reduction of serum plasma cholesterol [6]. Dietary proteins with low ratios of methionine–glycine and lysine–arginine, such as soy and fish proteins, also show a hypocholesterolemic effect in human body [130]. Contrary to these findings, biopeptides derived from bovine caseins tend to increase the blood cholesterol level, possibly owing to high ratios of methionine-glycine and lysine-arginine [6]. Information about the hypocholesterolemic effect of protein hydrolysates is limited; therefore, further investigations are required to identify the components or active peptide segments of protein hydrolysates. Table 3 shows several hypocholesterolemic peptides obtained from different food sources.

#### 3.3.1. Milk-Derived Bioactive Peptides

Nagaoka et al. [137] introduced hypocholesterolemic peptide IIAEK from β-lactoglobulin. They evidenced, using Caco-2 cells study, that the hypocholesterolemic action of β-lactoglobulin tryptic hydrolysate (LTH) is justified by preventing the absorption of cholesterol, and indicated higher hypocholesterolemic activity than the synthetic medicine in animal model [137]. It was also reported that dipeptide EK was significant for the hypocholesterolemic function of IIAEK. In fact, dipeptides contain a C-terminal lysine, which are important to identify a hypocholesterolemic peptide [137]. Morikawa et al. [138] investigated the hypocholesterolemic activity of dipeptides with a C-terminal lysine, using the evaluation of CYP7A1 mRNA level, and then proved that DK, EK, and WK dipeptides can increase the CYP7A1 mRNA level in HepG2 cells [139].

#### 3.3.2. Meat-Derived Bioactive Peptides

Papain-hydrolyzed pork meat was studied for cholesterol-lowering effect by Katsuda et al. [140] in rabbits fed a cholesterol-enriched diet. It was shown that the plasma and liver cholesterol concentrations were both significantly lower than in rabbits fed untreated pork meat. They suggest that peptides produced by papain-hydrolysis of pork meat have a hypocholesterolemic activity through their interference with the steroid absorption process.

#### 3.3.3. Marine-Derived Bioactive Peptides

The edible muscle part of freshwater clam after hot water extraction as value-added by-product was introduced as reference for producing hypocholesterolemic peptides by Lin et al. [136]. Hydrolysates obtained from pepsin were fractionated gel filtration and purified by RP-HPLC and subjected to amino acid sequence analysis. Two peptides VKP and VKK were identified as hypocholesterlemic peptides from the freshwater clam hydrolysate. Thus, freshwater clam hydrolysate can be used as functional ingredient in foods to prevent hypercholesterolemia. A correlation between the hydrophobicity of protein hydrolysates and their capacity to attach to bile acids was shown, indicating that peptides with a high bile acid-binding ability prevent the reabsorption of bile acids in the ileum and diminish blood cholesterol levels [136].

#### 3.3.4. Plant-Derived Bioactive Peptides

Soy protein hydrolysates and peptides can reduce blood total cholesterol levels more effectively than their parental protein. Bioactive peptides obtained from hydrolysis of soy glycinin, LPYPR, and IAVPGEVA were reported to reduce serum cholesterol [131]. In an early study, it was shown that soy peptide hydrolysates reduce LDL cholesterol levels by an average of 5.7%, and even a greater reduction (12.4%) was achieved in triglyceride levels [141]. Later, Pak et al. [142] investigated the hypocholesterolemic properties of globulins from soy beans. The bioactive peptide Leu-Pro-Tyr-Pro-Arg was isolated from soy-peptide glycinin. The hypocholesterolemic activity of this peptide was found to be higher than that of native soy peptide. It is possible that the hypocholesterolemic activity of peptides is owing to direct bile acid and neutral sterol binding in the intestine. Bioactive peptides extracted from chia seeds have been shown to have hypocholesterolemic function by inhibiting cholesterol homeostasis [143]. Furthermore, it has been documented that hydrophobic bioactive peptides containing leucine, tryptophan, and tyrosine have better hypocholesterolemic activity [144]. The peptide GCTLN from cowpea bean protein hydrolysates obtained from pepsin followed by pancreatin showed cholesterol-lowering effect. This peptide could attach the HMG-CoA reductase and inhibit the enzyme and reduce cholesterol micellar solubilization in vitro [145].

## 4. Application of Bioactive Peptides in Food Technology and Food Biosafety

There is an increasing awareness and interest among consumers for the importance of bioactive peptides as health-promoting ingredients in the diet. Enzymatic and acid/alkaline hydrolysis, microbial fermentation, or chemical hydrolysis are the conventional methods commonly used for the extraction and production of bioactive compounds [4,146]. These methods are usually accompanied by a membrane separation, a suitable technology that includes nanofiltration, and ultrafiltration modules, which are commercially available for industrial scale separation and purification of peptides based on their specific Mw range [104,147]. This critical step, which supposes percentages up to 70% of the peptide production costs, is followed by a purification step necessary for the production of bioactive peptides for food or nutraceutical applications [75].

The advantages associated with enzymatic hydrolysis make it the most widely used technique. Among them, its high specificity and the use of moderate operating conditions stand out, as well as the fact that it lacks toxicity. Something similar happens with microbial fermentation, which even avoids the high costs associated with the employment of enzymes. In contrast, the low yield of peptide generation of both methods and the low specificity of the peptides obtained make it necessary to explore alternative technologies [148].

High hydrostatic pressure processing (HHP), microwave-assisted extraction (MAE), ohmic heating, pulsed electric fields (PEFs), subcritical water extraction (SWE), and ultrasound-assisted extraction (UAE) are being explored for the production of bioactive peptides. Many of them can even be combined with hydrolysis in the form of pre-treatment to increase the yield in obtaining small Mw bioactive peptides [4,146]. However, despite the progress achieved with these innovative technologies, as they increase the extraction yields and the biological activity of the bioactive peptides by not affecting the composition and the structure their hydrolysate compounds, and they are more sustainable and environmentally friendly [4], there are still drawbacks that hinder their industrial application. This makes large-scale commercial production of bioactive peptides limited owing to the lack of proper industrial techniques and equipment.

Several bioactive peptides have already been commercially produced and exist in the international market, and there are functional food products enriched with such peptides. However, few studies have been conducted to evaluate the effect of bioactive peptides in real food matrices. There were attempts to incorporate the bioactive peptides into fermented dairy products at the end of the fermentation process in a pH range of 4.25–4.5 to produce functional and health-promoting products [149]. Przybylski et al. [150] applied the peptide TSKYR as a preservative in minced meat. This peptide, isolated from bovine hemoglobin by enzymatic hydrolysis with pepsin, allowed to decrease lipid oxidation during storage period as effectively as BHT (0.7 mg MDA/kg meat).

However, there are problems related to the direct application of bioactive peptides in food. Stability issues such as degradation and possible molecular interaction of the peptides with food components may decrease their bioactivity. Food processing conditions may also adversely affect the bioactivity of peptides. Moreover, some peptides interfere in the sensory attributes of food owing to a possible bitter taste. To overcome these effects, bioactive peptides or protein hydrolysates can be treated with stabilizing carriers. Several studies suggested that encapsulation of antimicrobial [151] and antihypertensive peptides [152,153] in liposomes has been suggested to overcome the problems related to the direct application of the peptides in food, as it can keep the structural integrity and functionality of peptides in food matrices [154,155].

**Figure 3:**
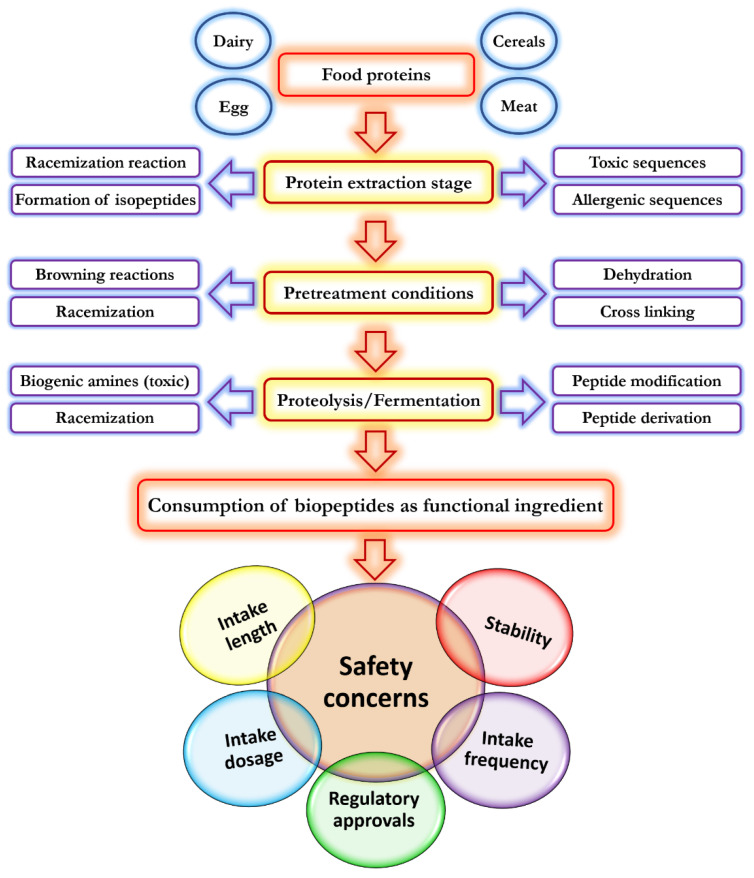
Safety concerns and hazards during preparation, storage, and consumption of bioactive peptides.

In addition, there are concerns about the safety of peptides in food application, as there is a possibility to form allergenic or toxic peptides from their parental proteins [156]. Moreover, hazardous components can be produced during protein pre-treatment and extraction processes to produce bioactive peptides [156]. Despite the fact that a large number of bioactive peptides are being isolated from food protein sources, most of these peptides are not consumed by humans yet, thus they cannot be considered safe. This is related to the fact that it is still not clear whether the bioactive peptides obtained from the food grade enzymes are safe. Various peptides are produced using non digestive enzymes and different substrates, or process conditions that do not exist in the human digestion process [157]. Therefore, the knowledge about the possible toxicity of bioactive peptides, and thus their safety aspects, is still limited. In this regard, important parameters such as peptides intake dosage, frequency, and length of administration have to be taken into account. Figure 3 demonstrates safety concerns and hazards during preparation, storage, and consumption of bioactive peptides.

In studying the safety of bioactive peptides, the following challenges must be understood [156]: food proteins are susceptible to change, leading to the creation of toxic or allergenic peptides; formation of undesirable compounds due to the highly sensitive nature of amino acids to the environmental and hydrolysis conditions; formation of derivate compounds such as nitrosamines, acrylamide, and biogenic amines as a result of interactions of peptides with food matrix during processing and storage; it is still not clear whether other food bioactive compounds like secondary metabolites can affect the safety of bioactive peptides or not; and bioactive peptides are considered beneficial only in appropriate doses, and their consumption at higher doses for long-term period in sensitive and allergic population may cause serious adverse effects.

## 5. Conclusions

This review focused on several food proteins-derived bioactive peptides that are potential modulators of various regulatory reactions in the human body. Physiologic attributes of bioactive peptides including antioxidant, ACE-inhibitory, and hypocholesterolemic activities were discussed. Bioactive peptides also show functional properties in food applications including water holding, foaming, emulsifying, and solubility properties. Therefore, functional foods and nutraceuticals can be developed by incorporating biologically active peptides into their formulation. However, it should be noted that the functionality and biological activities of the bioactive peptides are influenced by the conditions of the hydrolysis process. Moreover, peptide structure and its amino acid sequence can affect their physiological and therapeutic properties. In addition, it is also necessary to take into account that there are safety concerns about the food application of these peptides, as there is a possibility to form allergenic or toxic peptides from their parental proteins. Moreover, hazardous components can be produced during protein pre-treatment, extraction, enzymatic hydrolysis, or fermentation processes, as well as during storage. Although new techniques are necessary to retain the stability and biological activity of peptides in different food matrices, it seems that different techniques of encapsulation could resolve this problem. Thus, further studies are needed on the safety and quality of the foods containing bioactive peptides, and to develop modern techniques that make it possible to enrich active peptide from food protein and facilitate the industrial-scale production of these peptides.

## Figures and Tables

**Table 1:** Antioxidant activity of bioactive peptides obtained from different protein sources.

Protein Source	Enzyme	Peptide Amino Acid Sequence	Ref.
*Arthrospira platensis* protein	Alcalase	VTAGLVGGGAGK	[39]
Brewers’ spent grain	Alcalase	IPY and LPY	[40]
Cotton seed	Alcalase	YSNQNGRF	[41]
Rice dreg protein	Alcalase	GDMNP and LLLRW	[42]
Porcine liver	Alcalase	ALFQDVQKPSQDEWGK, APAAIGPYSQAVLVDR, FLANVSTVLTSK, FLEQQNQVLQTK, LGEHNIDVLEGNEQFINAAK, REATQPEVDTTLGR	[32]
Porcine liver	Alcalase, bromelain, flavourzyme, and papain	ALFQDVQKPSQDEWGK, APAAIGPYSQAVLVDR, GLNQALVDLHALGSAR, LSGPQAGLGEYLFER	[43]
Cheddar cheese	Microbial protease (*Lactobacillus helveticus*)	EMPFPK, KEMPFPK, SDIPNPIGSENSEK	[44]
Potato waste	Pancreatin and Amano-P	FGER, FDRR, FGERR	[45]
Rhizome proteins of ginger	Pepsin and trypsin	VTYM	[46]
Wheat germ	Pepsin	KELPPSDADW	[3]
Jackfruit seed	Trypsin	VGPWQK	[47]
Rapeseed	Alkali protease	WDHHAPQLR	[48]
Myosin, Spanish dry-cured ham	Pancreatin	SNAAC	[49]
Jinhua dry-cured ham	Pepsin-trypsin	LPGGGHGDL, LPGGGT, KEER	[50]
Bovine casein	pepsin	YFYPEL	[51]
β-lactoglobulin	Corolase PP	WYSLAMAASDI	[52]
Whey protein concentrate	Alcalase	WYSL	[53]
Pig bone collagen	Alcalase and neutrase	AGPAGPAGAR, AGPHGPPGKDGR, GPAGPHGPPGKDGR	[54]
Rice bran	papain and trypsin	VAGAEDAAK, AAVQGQVEK, GGHELSK, CQHHHDQWK	[55]

**Table 2:** Antihypertensive activity of bioactive peptides obtained from different protein sources.

Protein Source	Enzyme	Peptide Amino Acid Sequence	Ref.
Mung bean	Alcalase	KDYRL, VTPALR, KLPAGTLF	[98]
Brewers’ spent grain		ILDL, and ILLPGAQDGL	[40]
Mung bean	Bromelain	LPRL, YADLVE, LRLESF, HLNVVHEN, and PGSGCAGTDL	[99]
Brewers’ spent grain	Flavourzyme	IPLQP and LPLQP	[100]
Rhizome proteins of turmeric	Pepsin and trypsin	CGVGAA, DVDP, and CACGGV	[46]
Rhizome proteins of ginger		VTYM	[46]
Wheat germ	Proteinase K	SGGSYADELVSTAK	[2]
*Arthrospira platensis* protein	Trypsin	PTGNPLSP	[39]
β-lactoglobulin	Thermolysin	LDTDYKK	[59]
Buffaloe skim milk	Papain, pepsin, and trypsin	FPGPIPK IPPK QPPQ	[101]
Trevally (*Pseudocaranx* sp.)	Bromelain	AR, AV, and APER	[102]
Haeckel (box jellyfish) venom	Pepsin	ACPGPNPGRP	[103]

**Table 3:** Hypocholesterolemic activity of bioactive peptides obtained from different protein sources.

Protein Source	Enzyme	Peptide Amino Acid Sequence	Ref.
Soy protein	Alcalase	WGAPSL	[132]
	Trypsin and pepsin	IAVPGEVA, IAVPTGVA, and LPYP	[133]
Bovine milk β-lactoglobulin	Trypsin	IIAEK	[134]
Soybean glycinin		VAWWMY	[135]
Freshwater clam (*Corbicula fluminea*)	Protamex	VKP, VKK	[136]
Soybean		YVVNPDNDEN, YVVNPDNNEN	[132]

## Data Availability

The data presented in this study are available on request from the corresponding authors.
